# Augmented-reality template guided transorbital approach for intradural tumors

**DOI:** 10.3171/2021.10.FOCVID21172

**Published:** 2022-01-01

**Authors:** Walter C. Jean, Kenneth D. Sack, Andrew R. Tsen

**Affiliations:** 1 Division of Neurosurgery, Department of Surgery, Lehigh Valley Health Network, Allentown, Pennsylvania; and; 2 Department of Neurological Surgery, George Washington University, Washington, DC

**Keywords:** augmented reality, transorbital approach, transpalpebral

## Abstract

For “minimally invasive” approaches to a deep-lying skull base lesion, the bone opening must be precisely placed and adequately wide to accomplish the surgical goal. Surgical rehearsal in virtual reality (VR) can generate navigation-integrated augmented reality (AR) templates to ensure precise surgical openings.

In this video, the authors used AR templates for the transpalpebral, transorbital approach for intradural tumors. VR renderings of patient-specific anatomy were used in surgical rehearsal. The optimal openings were saved and, at surgery, projected into the eyepiece of the navigation-tracked microscope. The template enhanced the planning of the incision and soft-tissue exposure and guided the drill toward the target.

The video can be found here: https://stream.cadmore.media/r10.3171/2021.10.FOCVID21172

## Transcript

This video demonstrates the technique of using augmented reality templates to guide the transorbital approach for intradural tumors.^1–3^

For a “minimally invasive” approach to a deep-lying skull base lesion, the bone opening must be small yet provide adequate exposure to the surgical target. Surgical rehearsal in virtual reality (VR) can reveal the nuances of patient-specific anatomy and simultaneously generate navigation-integrated augmented reality templates to ensure precise surgical openings.^4,5^

To do this, three-dimensional VR renderings of patient-specific anatomy were used in surgical rehearsal. The optimal openings were saved as VR files and, at surgery, projected into the eyepiece of the navigation-tracked microscope. The templates enhanced the planning of the incision, soft-tissue exposure, and guided the precise bony removal to the target. Here is our demonstration of the technique.

### 1:13 Beginning of Case 1.

The first patient was a 60-year-old woman who presented with mental status changes. She had a few days of headache, and while playing cards, the other players noted she was confused and had some facial asymmetry. She was neurologically intact on exam.

### 1:25 Imaging Findings of Case 1.

Her imaging studies showed a cystic tumor of the right temporal lobe and the differential diagnosis was cystic metastasis and primary glioma.^6^ A CT of the chest, abdomen, and pelvis was performed and it was negative. The plan was for a transpalpebral, transorbital approach for resection of this tumor.

Surgical rehearsal in virtual reality was useful to examine the exposure of the approach, as well as prepare a template which we will use to guide the surgical opening.

### 1:48 Surgical Rehearsal of Case 1.

The MacCarty burr hole is useful and marked in blue. The lateral orbital rim is removed with intention to reattach after surgery. Both wings of the sphenoid and the lateral orbital wall are removed. The orange marker denotes the most anterior tip of the tumor on the dura. This exposure should be sufficiently wide for the surgical goal to be safely achieved. We will save this as a file to be used intraoperatively.

### 2:16 Incision of Case 1.

The transpalpebral approach was used for this approach and the incision was made along a lid crease. The dissection is then taken between the layer of the orbicularis oculi and the septum. Care must be taken that the septum is not violated. The supraorbital ridge and the lateral orbital rim are both exposed. And the periorbital is carefully protected as well as dissected from the bone. The lateral orbital rim is then removed, just like in VR.

### 3:00 AR Template Projected Onto Field of Case 1.

Here you see the template projected as an AR overlay via a navigation-tracked microscope. And it guides us for the drilling of the sphenoid wings. The dura at the temporal tip was readily exposed, and with the removal of the last bit of the lesser wing that separates the anterior and middle fossa, we are ready to open the dura of the temporal tip. But before that, we brought in the AR template once again to make sure we have adequate exposure to resect the tumor.

Now, we open the dura. The tumor is directly underneath, and the tumor resection proceeds as per routine. Once the cyst is reached, we know we’ve reached the posterior and superior extent of the tumor. For closure, the lateral rim is reattached and a Medpor implant is tucked underneath the temporalis. Imaging confirms a full resection. Looking at the inside of the skull, the blue portion with the orange marker shows the extent of the bone drilling. We did encounter a CSF leak postop, stopped by lumbar drainage. And the patient was satisfied with the cosmetic result. She was back to her neurological baseline after surgery. The tumor was a GBM and she went on to receive chemoradiation.

### 4:01 Beginning of Case 2.

The second patient was a 41-year-old woman who presented with syncopal episodes. Her cardiac workup was negative and the episodes resembled seizures. She was neurologically intact.

### 4:12 Imaging Findings of Case 2.

Her MRI showed a left-sided, juxtacavernous meningioma with temporal lobe edema. The surgical goal was resection to hopefully eliminate the tumor and the associated brain edema. And a transorbital approach was selected, as well as a transpalpebral incision.

Rehearsal in VR was performed similar to the first patient, and templates were generated. This is the superficial augmented reality template showing the planned lateral orbitotomy and the deep AR template showing the bone removal of the lateral orbital wall and sphenoid wing.

### 4:40 Intraoperative Video of Case 2.

After the transpalpebral incision and soft-tissue opening just like the last case, here we are drilling the deep template, with the purple line showing the anterior edge of the tumor.

The tumor is projected as an AR mesh confirming that the bony opening is sufficient now. The green marker is the root of the anterior clinoid process, and we have a rare glimpse of the meningo-orbital band from an intraorbital perspective. The meningo-orbital band is cut and a partial anterior clinoidectomy is completed to give sufficient access for tumor resection.

### 5:14 Peeling of Lateral Cavernous Sinus Wall.

The peeling of the lateral cavernous sinus wall is again done from an intraorbital angle, which is unusual. Once the dura is opened, the tumor is stepwise debulked, mobilized, and then removed until complete.

The lateral rim is replaced at the end of the procedure and the radiographic, neurological, and cosmetic results were all very satisfactory. She was at her neurological baseline after surgery. The tumor was a WHO grade I meningioma.

### 5:52 Summary.

In summary, rehearsal in VR is a valuable tool for trainees and surgeons alike^4,5^ and, coupled with AR, allows for more precision, efficiency, and control during surgery. We demonstrated the application of AR templates to ensure that minimally invasive openings were precisely placed and provided adequate exposure to achieve the surgical goal. In this scheme, the surgeon is no longer using navigation to get his or her bearings. Instead, the surgeon is using AR-enhanced navigation to duplicate a plan that is known to work. This is a fundamental paradigm shift.

## Disclosures

Dr. Jean serves as a consultant for Surgical Theater LLC.

## Author Contributions

Primary surgeon: Jean. Editing and drafting the video and abstract: all authors. Critically revising the work: Jean, Tsen. Reviewed submitted version of the work: Jean, Tsen. Approved the final version of the work on behalf of all authors: Jean. Supervision: Jean.
